# Turning over New Leaves

**Published:** 1890-08-02

**Authors:** 


### Turning Over New Leaves.

### Faith Cures.*

Here is a little book of 190 pages, and, although we are constrained to admit that its publication in this "so called Nineteenth Century" is an anachronism, we think that those who wish to study the Faith Cure can hardly do better than run through the one-and-twenty rather pleasantly written chapters of which it is composed. We concede at once that Mr. Gliddon writes in perfect good faith, and, with much candour, warns his readers that some of the cures may have been of imaginary diseases. Here he will find that all readers of The Hospital will agree with him; but leaving out of account these imaginary diseases, we are convinced, and we presume we have the whole band of physicians and surgeons with us, that if the element of hope and belief in a cure can be instilled into the patient suffering from disease, recovery is much more likely to occur in curable diseases, and improvement in incurable ones, than where hope is absent. Great is the effect of the mind on the body, and it would seem to matter little how that hope is stirred up, the result will be the same.

Perhaps the most useful parts of the book under notice are the historical ones—that is, those sections dealing with the Peculiar People, the Salvation Army, the Bethshan Home, and the Catholic Apostolic Church. To this extent the little volume may be cordially recommended; but when we examine the evidence of the cures we feel that such evidence is wholly inconclusive, and cannot be accepted. It is beyond doubt that many of the so-called nervous affections are in great part imaginary, and "cures" are not unlikely to follow the exercises mentioned. In other cases, again, a distinction must be drawn between recovery and belief in recovery. It may be useful to give in detail one of the "cures," for our readers' edification and judgment: A man who had some (not stated) disease of the foot and leg, had been told by a "well-known surgeon" that his foot might be taken off, and then his leg, and yet the disease would be untouched. He had suffered from it for seven years. What could the disease have been? However, he went to the London Bethshan, and at the first meeting he left his crutch and cane behind him. "The second time he was much better than the first; and after the third he walked freely without help, dancing his way the last fifteen yards to the cab." We are told that some of the cases were of a "decidedly striking character," and that the author "was struck with the wonderful variety of diseases whose victims have declared that they have found relief." We read of bronchitis, spinal paralysis, epilepsy, neuralgia, heart disease, consumption, numerous tumours, blindness, lameness, sciatica, rupture, nervous debility, liver complaints, deafness, scrofulous swellings, curvature of the spine, spasmodic asthma, withered arm, cancer, insanity, rheumatic gout, polypus, cataract, varicose veins, dyspepsia, flatfoot, whooping cough, Bright's disease, stammering, measles, burns, blood poisonings, cholera, pleurisy, piles, diphtheria, scarlet fever, corns, chilblains, and skin diseases. We do not wonder at Mr. Gliddon being struck by this list, for we were struck by it ourselves, and we could not reconcile it with the hundreds of hospitals and thousands of doctors who thrive in London, until we found that our guide had naïvely added that, "in many instances the cures were incomplete at the time when the testimony was given."

In the second part of Henry VI. the great magician Shakespere tells us of a sudden cure of lameness and blindness, the application of a horsewhip being the treatment employed; and there could be no doubt that the cure resulted from the treatment.

The old stories which Mr. Gliddon rakes up of the daughter of the Emperor Valeus and of Aspasia are no evidence whatever. They merely show that such things were credited in those days, not that they really happened.

The book contains an appendix in which are given the accounts of seventy cases of cures. Some of these read wonderfully like the testimonials in favour of quack medicines, and we are specially cautioned that "some of the witnesses may have been mistaken in supposing themselves to have been afflicted with the particular ailments named." To which we echo "very likely." We cannot resist quoting one or two of these specimens. "Blind Mary, of Preston, near Hitchin, bore testimony, at 3, Highbury Place, to her healing. She had been totally *blind* for sixteen years, *dumb* for three years, one side of her body had been paralysed for twelve years, and she had not moved herself in bed for many years. She was healed by faith when about 58 years of age."

"Mr. Brown, of Barking, said at a Bethshan meeting in February, 1885: 'I suffered from epileptic fits for years. My wife came to Bethshan, and telling me about it I said, "It is the work of the devil." But she came here again with a diseased throat, not having spoken above a whisper for three months. That same night she sat down to the piano and sang to me.'" Dominie Sampson would have said "prodigious!" but we end by congratulating Blind Mary and Mr. Brown on their recoveries.

* "Faith Cures, their History and Mystery." By Aurelius J. L. Gliddon. (London: *Christian Commonwealth* Publishing Company, Limited.) Price 2s.

